# Cancer T-cell therapy: building the foundation for a cure

**DOI:** 10.12688/f1000research.27217.2

**Published:** 2020-12-22

**Authors:** Alexander Kamb, William Y. Go

**Affiliations:** 1 A2 Biotherapeutics, Agoura Hills, California, 91301, USA

**Keywords:** CAR, TCR, cancer, mechanism of action, clinical translation, innovation

## Abstract

T-cell cancer therapy is a clinical field flush with opportunity.  It is part of the revolution in immuno-oncology, most apparent in the dramatic clinical success of PD-1/CTLA-4 antibodies and chimeric antigen receptor T-cells (CAR-Ts) to cure certain melanomas and lymphomas, respectively.  Therapeutics based on T cells ultimately hold more promise because of their capacity to carry out complex behaviors and their ease of modification via genetic engineering.  But to overcome the substantial obstacles of effective solid-tumor treatment, T-cell therapy must access novel molecular targets or exploit existing ones in new ways.  As always, tumor selectivity is the key. T-cell therapy has the potential to address target opportunities afforded by its own unique capacity for signal integration and high sensitivity.  With a history of breathtaking innovation, the scientific foundation for the cellular modality has often been bypassed in favor of rapid advance in the clinic.  This situation is changing, as the mechanistic basis for activity of CAR-Ts and TCR-Ts is backfilled by painstaking, systematic experiments—harking back to last century’s evolution and maturation of the small-molecule drug discovery field.   We believe this trend must continue for T-cell therapy to reach its enormous potential.  We support an approach that integrates sound reductionist scientific principles with well-informed, thorough preclinical and translational clinical experiments.

*If you have built castles in the air, your work need not be lost; that is where they should be. Now put the foundations under them.*

Henry David Thoreau, *Walden* 1854

## T-cell therapies are the future of oncology

It is astounding how the contents of the typical pharmacy have changed over the last 100 years. A century ago, pharmacists stocked their shelves with aspirin, opiates, mercury, arsenic, magnesium sulfate, iodine and a few other substances of legitimate medical value ( Pharmacopoeia of the US, 1907). Since then, hundreds of small-molecule drugs, dozens of recombinant antibodies, and even a few nucleic acid therapeutics have been proven by rigorous scientific and clinical studies to treat a wide variety of human ailments. It is likely, however, that for a large number of patients yet to enjoy effective remedies for their disease, including cancer, cell therapy will ultimately provide the solution. 

This prediction follows from the inherent strengths of cells as therapeutic entities. T cells, for example, are honed by evolution to execute numerous complex biological functions, among them identification and elimination of infected or damaged tissue ( Janeway *et al.*, 1999). They have tremendous natural advantage over other therapeutic modalities that are often limited to a single activity: binding to other molecules. Simple binding behavior may be sufficient to trigger salutary physiological changes and, indeed, there are many examples. However, the limitations imposed by having only hundreds of atoms like small molecules, or even thousands like antibodies, is evident. T cells, on the other hand, are composed of thousands of different molecules, prewired by evolution to work in concert to accomplish tasks of extraordinary complexity ( Janeway *et al.*, 1999). Specific killing is one of the simpler cellular behaviors, and is therefore among the first successful achievements of T-cell therapy, exemplified by three CD19-targeting chimeric antigen receptor T-cells (CAR-T cells) registered or close to registration ( Abramson, 2020; Neelapu *et al.*, 2017; Neelapu *et al.*, 2020a; Schuster *et al.*, 2019). The next frontier for engineered T-cell therapy is solid tumors, which pose additional challenges. Perhaps most dramatically, infused T-cell therapeutics directed against solid tumors must extravasate to reach their targets, targets that may be present on a subset of vital normal tissues as well. But cells have a second huge advantage as a therapeutic option: they can be readily manipulated with genetic alterations to augment or suppress their natural behaviors. The methods to do this are now routine and are improving with the advent of newer technologies such as CRISPR/Cas9 ( Cong *et al.*, 2013; Jinek *et al.*, 2012). Combined with cellular reprogramming technologies, the possibilities to modulate natural cell properties or even create emergent ones are wide open ( Takahashi & Yamanaka, 2006; Yu *et al.*, 2007). T cells are naturally endowed with the attributes of (i) outstanding sensitivity, able to detect a handful of molecules on a cell surface; (ii) multivariate signal integration, permitting them to react to different environments and discriminate among a variety of cell types; and, (iii) the capacity to proliferate. These traits are exactly those needed to overcome obstacles posed by solid tumor therapy.

## We need to build a robust mechanistic foundation

To overcome the obstacles to solid tumor therapy, we must first recognize certain facts. A hallmark of the T-cell therapy field is striking innovation, with towering figures such as S.A. Rosenberg who has spent 40 years spearheading the clinical use of T cells in cancer ( Fisher *et al.*, 1989; Yron *et al.*, 1980). Others, including G. Gross and Z. Eshhar (CAR), M.R. Roberts and M.H. Finer (Gen2 CAR), and V.D. Fedorov and M. Sadelain (iCAR) have designed robust novel receptors that can substitute for, or extend, T-cell receptor (TCR) function ( Fedorov *et al.*, 2013; Gross *et al.*, 1989; Roberts *et al.*, 1994). Still others have made substantive contributions to understanding, design and development of next-generation CAR-Ts; for example, C. June and P. Greenberg (see for review Guedan *et al.*, 2019).

**Figure 1.**
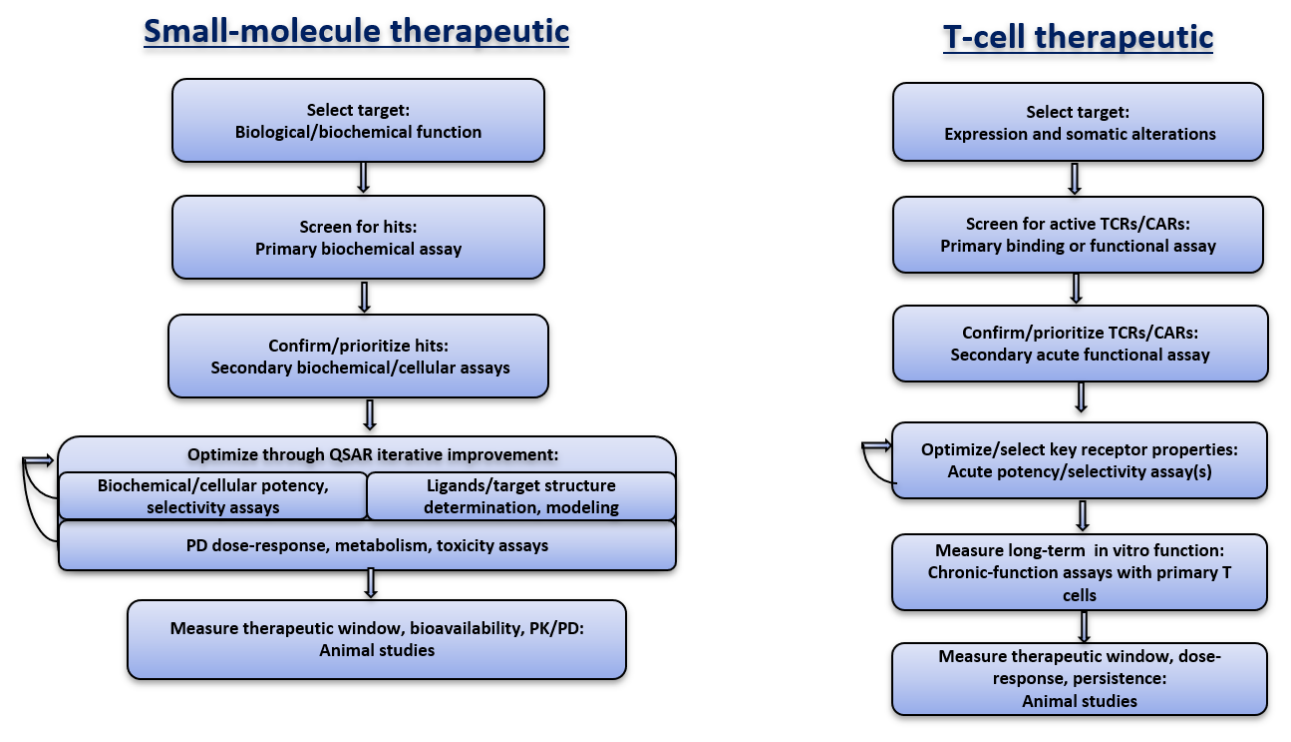
Flow scheme of drug discovery, comparing small-molecule to T-cell therapeutic discovery. QSAR, quantitative structure-activity relationship. The goal is to control variables and improve the predictability of substantive advances.

Notwithstanding the innovation and clinical success, the field lacks a strong foundation of mechanistic understanding. For example, there is not a broadly accepted model that explains key behavior of TCRs with respect to sensitivity and selectivity toward their ligands, peptide major histocompatibility complexes (pMHCs). CAR signaling, though understood in outline, also lacks important details (see for review Courtney *et al.*, 2018; Nerreter *et al.*, 2019). These gaps impede progress in areas that need to be addressed so that solid tumors can reliably and predictably be treated. It is instructive to draw an analogy with small-molecule drug discovery, a field that developed over the 20 ^th^ century from rudimentary industrial processes to a highly sophisticated discipline of quantitative structure-activity relationships based on structural chemistry, computational modeling, and pharmacodynamic analysis *in vitro* and *in vivo* ( Figure 1).

As an emerging field, engineered T-cell therapy is not on a similarly solid footing. The standard suite of *in vitro* assays is crude when compared to those used in modern small-molecule or antibody optimization laboratories. Assays that vary effector:target ratios are convenient, but have high background and poor dynamic range. They are typically insensitive and subject to conflation of important biological variables; for instance, T cell proliferation and cytotoxicity as well as target-cell proliferation over time ( Rossi *et al.*, 2018). Primary human T cells are heterogeneous and cumbersome to grow, with considerable donor-to-donor variability; and the relationship between them and model cell lines, such as Jurkat, is not well understood ( Salter & Creswell, 1986). Murine cancer models must also trade off tractability with relevance, and have some obvious *prima facie* weaknesses. Assays of therapeutic efficacy and safety in murine models are notoriously unpredictive for clinical behavior ( Kamb, 2005). These deficits apply to small- and large-molecule therapeutic discovery. In immuno-oncology specifically, even the best models use syngeneic grafts that do not originate in the host and, though matched at MHC, contain hundreds of nonsynonymous mutations and elicit immune response ^1^. Many experiments employ chimeric murine models with a complicated mixture of murine and human immune components (e.g., humanized murine models, patient-derived xenografts). The human and mouse components of these chimeras, e.g., IL-2 and IL-2R, do not mesh perfectly ( Nemoto *et al.*, 1995). These models have utility and are chosen for practical reasons, but they are often regarded as decisive in selection of clinical candidates because of presumptive experimental supremacy. In our view this is specious. The ultimate destination of a clinical candidate is the complex milieu of the human body and specifically the tumor microenvironment. But understanding the steps that must occur, one by one, to achieve a successful outcome in the clinic should not be dismissed as irrelevant just because they are studied outside the system biology of a human body. *In vivo* experiments should be used and interpreted judiciously in the context of robust *in vitro* data. As a T-cell therapy example, simple xenograft models demonstrate that therapeutic function is compatible with the environment of a mammalian body; nothing more, but nothing less.

Referencing small-molecule discovery again, the most successful efforts have involved deliberate construction of a mechanistic picture; from biochemical assays, through cell-based assays, to cautiously interpreted *in vivo* testing of pharmacodynamics. A clear example is the history of imatinib’s discovery ( Buchdunger *et al.*, 1996). T-cell therapy would benefit from adoption of this approach to control as many of the variables as possible within a reasonable timeframe of drug discovery. Only then can the predictability of the discovery process improve to the point needed to address the challenges of solid tumor therapy. If we wish to continue to innovate and not settle for incremental advances to CD19-directed therapies where there are currently hundreds of ongoing clinical trials for an unmet need, now estimated at ~6,000 deaths/year in the US, we must improve the mechanistic understanding and economical testing of candidate therapeutics. Otherwise, the opportunity costs will be enormous.

## The acute shortage of solid-tumor drug targets: targeting genetic gains and losses

Selectivity is the supreme challenge of oncology. At the genetic level, a tumor differs on average at ~10,000 nucleotide positions from the normal tissues from which it arose—less than 0.01% of the human genome ( Vogelstein *et al.*, 2013). In contrast, siblings differ by about 10 million nucleotides. Perhaps even worse from a conventional therapeutic perspective, very few of these genetic changes are shared among a significant percentage of cancers. Only a handful of mutations, such as mutant KRAS and P53, occur at frequencies above 5% of cancers. The vast majority are private mutations unique to each tumor. For decades, drug discoverers have searched for “magic bullets” that can discriminate reliably among tumor and normal cells, with some success. Good examples include imatinib for chronic myeloid leukemia, which inhibits the Abl kinase, and rituximab, a CD20 antibody that mediates the destruction of B-cell lineage cells such as non-Hodgkin lymphoma ( Anderson *et al.*, 1997; Buchdunger *et al.*, 1996). Both these medicines are extremely effective within the subset of cancers they are designed to treat. In solid tumors, there are a few proteins, known loosely as tumor-selective antigens, whose expression is sufficiently limited in adult normal tissues that they continue to attract attention as possible cancer targets. These include CEA, MSLN, PSMA, and the MAGE family members ( Lu *et al.*, 2017; Parkhurst *et al.*, 2011).

In 2001 the complete human gene list of ~20,000 was defined, establishing a boundary for new discoveries. Cancer researchers have scoured this gene set for the last two decades with diminishing success, visible in the shrinking, overlapping group of cancer targets swarmed by academic research laboratories and pharma/biotech industry R&D organizations. We desperately need new options; and these will likely require utilization of known gene products in novel ways. Immuno-oncology offers prospects for doing so. The large majority of recurrent somatic mutations affect proteins expressed inside cells. Thus, it is necessary to overcome the barrier of the cell membrane that excludes antibodies and most other macromolecules to exploit somatic mutations as a source of selective cancer targets. The immune system has evolved the means to do so through the aegis of antigen presentation. Molecular complexes of major histocompatibility antigens bound to peptides derived from cellular proteins (pMHCs) give T cells a view of the internal contents of cells. Some of these pMHCs are likely the basis for PD-1 antibodies’ and tumor infiltrating lymphocytes’ (TILs) remarkable power to trigger tumor-specific killing by the immune system ( Chamoto *et al.*, 2020; Hinrichs & Rosenberg, 2014). pMHCs that contain mutant peptides are currently the intended targets for numerous investigational vaccines and T-cell therapy efforts to engineer or select neoantigen-reactive T cells ( Castle *et al.*, 2019; Ng *et al.*, 2019). The small number of recurrent mutations constrain the target options on this front. Though there are dozens—even hundreds—more private neoantigens, therapeutic exploitation of these via T cell engineering presents other challenges ( Ng *et al.*, 2019).

Loss of genetic material, rather than gain of somatic mutations, represents another opportunity to achieve absolute discrimination at the genetic level between tumor and normal cells. The most common form of genetic loss in cancer is loss of heterozygosity (LOH). An astonishing 20% of the genome in a typical cancer cell exhibits LOH. These LOH regions include loci that encode polymorphic surface antigens that can be recognized by T cells. Genetic loss is irrevocable and furnishes a basis for discrimination, provided a method can be devised to take advantage of LOH. The workings of a primordial branch of the immune system show the way. Natural killer (NK) cells, which evolved before the adaptive immune system, employ a system of signal integration that differentiates self from non-self by combining inputs from families of activating and inhibitory receptors ( Bryceson & Long, 2008). The logic of the NK system has been reproduced in an artificial circuit involving CARs ( Fedorov *et al.*, 2013). Versions of this basic circuit are capable in principle of utilizing LOH as a black-and-white difference between tumor and normal cells ( Hamburger *et al.*, 2020). Other approaches are under study, including transcriptional logic circuits and receptor masking ( Desnoyers *et al.*, 2013; Roybal *et al.*, 2016). These attempts to widen the target source for selective cancer targets to other targets, including neoantigens and LOH, are in their early stages, but they hold promise to dramatically increase the therapeutic options available for solid tumor patients.

## Additional challenges for T-cell therapy

The justifiable excitement around cancer T-cell therapy must be balanced with acknowledgement that many significant challenges remain beyond tumor-selective targeting. Difficulties in T-cell manufacturing and delivery to patients translate into high production costs and time-delays ( Fiorenza *et al.*, 2020; Locke *et al.*, 2020). Despite the technical hurdles, we view these issues as solvable through the iterative improvement cycles that are part of the standard practice of engineers. Efforts to automate, miniaturize and accelerate the production of autologous cells are underway ( Castella *et al.*, 2020). The opportunity to improve efficiency seems extremely attractive because the current doses of T cells range from 100 million to 100 billion cells—well beyond the number involved in a typical immune response in the body ( Gudmundsdottir *et al.*, 1999). Meanwhile, production methods for off-the-shelf allogeneic cell products have demonstrated early clinical success ( Neelapu *et al.*, 2020b). 

Perhaps more significant, efficacy to date in solid tumors is unimpressive and safety issues, either off- or on-target, continue to plague clinical programs ( Lu *et al.*, 2017; Norberg *et al.*, 2020; Parkhurst *et al.*, 2011). We believe these problems are also solvable. They will be addressed by biological solutions, as they are not generally the result of limits imposed by laws of physics and chemistry which constrain more mature modalities. Indeed, there are a myriad of levers to pull to improve T-cell therapy outcomes. In some respects, the opportunity set for improved design of T-cell therapeutics is so large, that the challenge is to prioritize and test the possibilities efficiently.

## An approach to future T-cell therapeutic discovery

We do not subscribe to the common view that human testing always trumps preclinical data, not because it is false in concept, but because it is problematic in practice. Variation in the clinic is typically large, the number of observations small, the expense high and timelines long ( Locke *et al.*, 2020; Silbert *et al.*, 2019). We believe that well-designed preclinical experiments, interpreted within a solid framework of pharmacology and biology, will greatly aid analysis of clinical results, and in the long run support translational innovation that saves lives.

**Figure 2.**
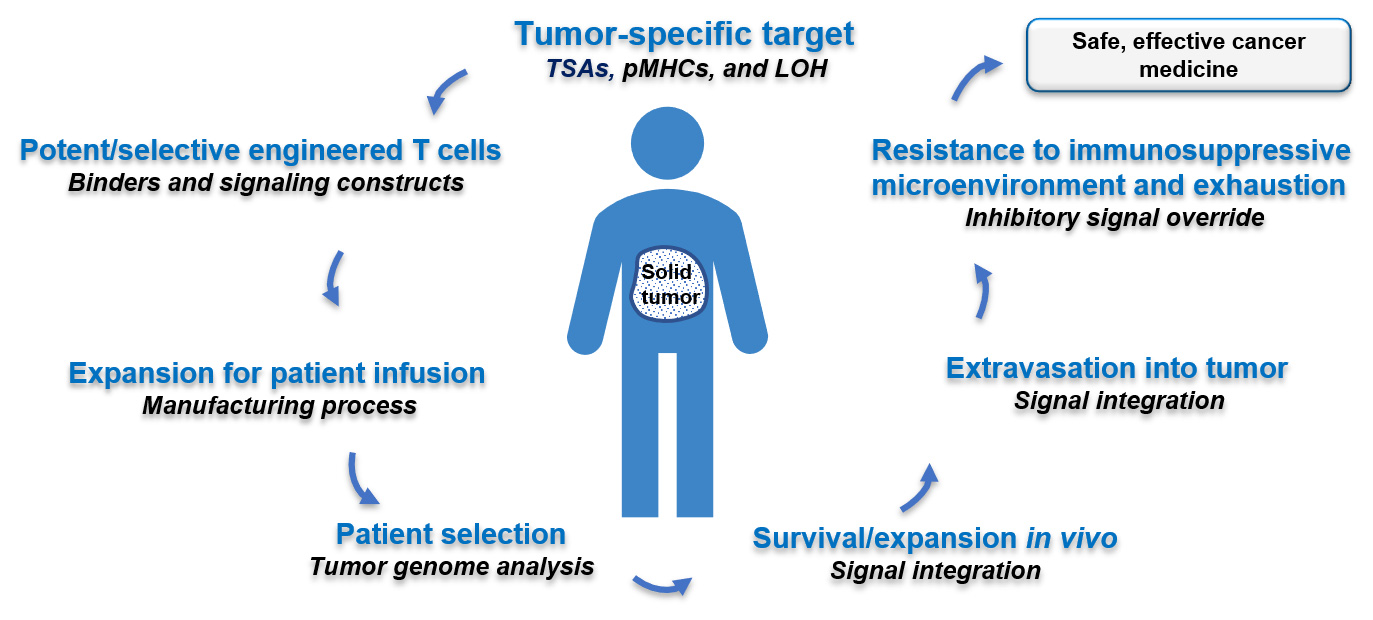
Cell therapy for solid tumors: step-wise requirements for success. This diagram illustrates the number and complexity of the steps required to achieve efficacy. Many of these steps can be studied *in vitro*; for others (e.g., extravasation), *in vitro* models are inherently problematic. TSA, Tumor Specific Antigen; pMHC, peptide-major histocompatibility antigen; LOH, loss of heterozygosity.

To this end, we propose a roadmap that begins by reducing the problem of solid tumor cell therapy into its components ( Figure 2). These components incorporate essential requirements for solid tumor cell therapy to achieve efficacy and safety, including that the engineered cells must: (i) survive in the body post infusion; (ii) migrate through the body’s tissues into the tumor microenvironment; (iii) overcome the potentially anti-inflammatory environment of the tumor; (iv) specifically recognize the tumor cells in a vast excess of normal cells; and, (iv) deliver a sustained cytotoxic blow sufficient to remove most, if not all, of the tumor bulk. These component activities can be parsed into scientific disciplines of biochemistry, pharmacology, cell biology, immunology, and tissue/organismal physiology. 

There are many potential differences between, for example, TCRs and CARs which have not been tested systematically, and the field would benefit from their thorough examination ( Table 1). It would be useful to have sufficiently large datasets to delineate the connection between tractable models and the more complicated preclinical systems, and ultimately, the clinic. In particular, we believe that quantitative assays that measure absolute sensitivity of receptors should be more widely employed, allowing direct comparisons among different targets and receptors. The collective time and expense on the one hand, and risk of irrelevant or non-robust results on the other, create significant overhangs for the field. Effort should be directed toward providing clear evidence to connect receptor properties to function, and T cell lines to primary cells. Given the potential importance of long-term survival and function of T cells for curative treatment of solid tumors, there is a pressing need for plausible *in vitro* models of chronic T cell activity. It is impractical to funnel large numbers of candidate receptors through *in vivo* models. This foundation-building work may not be glamorous, but is of great consequence and should be valued by scientific journals. Our strong view is that granting agencies should invest in foundation-building academic research, in part because shorter-term translational work is often attractive to the private sector. If the field as a whole invests to build the infrastructure and expertise of better preclinical models and larger datasets, and allocates time to define key mechanistic details prior to clinical testing, we believe the risks required to develop inventive, differentiated therapies will be rewarded with success.

**Table 1.** Potential differences among cell therapy targets, receptors, and regulation not yet rigorously tested by mechanistic data. Experiments to test many of these assumptions are underway.

Molecule	Specific attribute	Assumption	Basis
**Target**	**High tumor expression of** **target**	**Efficacy advantage**	**Higher density increases activation** **probability**
	**Solid tissue expression of** **target**	**Safety/tolerability** **challenge**	**No mechanism for tumor/normal** **discrimination**
**Receptor** **(CAR and** **TCR)**	**Avidity correlation with** **function**	**CAR>TCR**	**TCR known to have disconnects (e.g., pMHC** **antagonism)**
	**Target flexibility**	**CAR>TCR**	**TCR uses only pMHCs; CAR can target surface** **antigens and pMHCs**
	**Sensitivity**	**TCR>>CAR**	**TCR at the limit of sensitivity** **(1–10 pMHCs)**
	**Selectivity**	**TCR>>CAR**	**TCR evolves in body**
	**Tractable molecular** **engineering**	**CAR>TCR**	**TCR structure highly constrained**
	**Co-stimulation** **independence**	**CAR>TCR**	**Required for TCR activation**
	**Checkpoint resistance**	**CAR>TCR**	**TCR sensitive; e.g., PD-1 mAb therapeutic** **benefit**
	**Exhaustion susceptibility**	**CAR<TCR**	**CAR short-circuits regulators**

## Conclusion

The head of Novartis’ drug discovery organization, J. Bradner, reportedly expressed the opinion last year that “money and scientific resources are being poured into attempts to make incremental progress at a time when there is an urgent need for disruptive change” ( Usdin, 2019). We agree with this perspective, but would add that without proper investment in foundational understanding of the science and technology, efforts to innovate further engineered T-cell therapies are likely to bog down in frustrating unpredictability. Risk tolerance must be wedded to broad, deep preclinical datasets that enable better prediction of outcomes on the clinical frontier.

## Data availability

No data is associated with this article.

## Footnotes

^1^ https://www.criver.com/sites/default/files/resources/Whole-ExomeSomaticMutationAnalysisofMouseCancerModelsandImplicationsforPreclinicalImmunomodulatoryDrugDevelopment.pdf.
