# Supplementary material for: Cancer T-cell therapy: building the foundation for a cure
Source: F1000Res. 2020 Dec 22;9:1295. Originally published 2020 Nov 3. [Version 2] doi: 10.12688/f1000research.27217.2 (PMC7713893; doi:10.12688/f1000research.27217.2)
Supplement: Peer review history

# Peer review: Reviewer response for version 2

**DOI:** 10.5256/f1000research.31447.r76353

**Published:** 2021-01-04

**Authors:** Barbra J. Sasu

**Affiliations:** 1 Allogene Therapeutics, Inc.,, San Francisco, CA, USA

I think that the revised version makes the author's intent in writing the review much more clear and is a valuable perspective.  

 One small comment that I would suggest is changing the C. June and P. Greenberg mention: ‘next-generation CAR-Ts' to 'CAR-Ts and TCR-Ts’ to more accurately describe Greenberg's work.

Is the topic of the opinion article discussed accurately in the context of the current literature?

Partly

Are arguments sufficiently supported by evidence from the published literature?

Partly

Are all factual statements correct and adequately supported by citations?

Partly

Are the conclusions drawn balanced and justified on the basis of the presented arguments?

Partly

Reviewer Expertise:

T cell biology, CAR T

I confirm that I have read this submission and believe that I have an appropriate level of expertise to confirm that it is of an acceptable scientific standard.

# Peer review: Reviewer response for version 2

**DOI:** 10.5256/f1000research.31447.r76354

**Published:** 2021-01-04

**Authors:** John R. James, Muna Fuyal

**Affiliations:** 1 Warwick Medical School, University of Warwick, Coventry, UK; 2 School of Life Sciences, University of Warwick, Coventry, UK

The authors have done a good job responding to all the comments of the reviewers, and so we are happy to approve the article.

Is the topic of the opinion article discussed accurately in the context of the current literature?

Partly

Are arguments sufficiently supported by evidence from the published literature?

Partly

Are all factual statements correct and adequately supported by citations?

Partly

Are the conclusions drawn balanced and justified on the basis of the presented arguments?

Partly

Reviewer Expertise:

T cell signalling, signal transduction, reductionist approaches, Synthetic biology

We confirm that we have read this submission and believe that we have an appropriate level of expertise to confirm that it is of an acceptable scientific standard.

# Peer review: Reviewer response for version 2

**DOI:** 10.5256/f1000research.31447.r76355

**Published:** 2020-12-23

**Authors:** C. Glenn Begley

**Affiliations:** 1 BioCurate, Parkville, Vic, Australia

Thank you for noting my comments. I have nothing further to add.

Is the topic of the opinion article discussed accurately in the context of the current literature?

Partly

Are arguments sufficiently supported by evidence from the published literature?

Partly

Are all factual statements correct and adequately supported by citations?

Yes

Are the conclusions drawn balanced and justified on the basis of the presented arguments?

Yes

Reviewer Expertise:

Translational research - oncology

I confirm that I have read this submission and believe that I have an appropriate level of expertise to confirm that it is of an acceptable scientific standard.

# Peer review: Reviewer response for version 1

**DOI:** 10.5256/f1000research.30072.r74926

**Published:** 2020-12-02

**Authors:** C. Glenn Begley

**Affiliations:** 1 BioCurate, Parkville, Vic, Australia

This review highlights the success of the recent advances in immune-oncology focusing particularly on cellular therapies, and outlines some of the fundamental scientific criteria that have been ‘by-passed’ in moving into the clinic, but that will likely need to be understood to make the cell-therapy approach applicable to solid tumors.

 I note the valuable comments of James *et al.*, and in addition suggest:

1. As part of the “revolution in immune-oncology” seen with checkpoint inhibitors, CAR-T cells, the authors should acknowledge another ‘recombinant cellular therapy’ – oncolytic viruses.
2. The authors are appropriately critical of mouse models that “trade off tractability with relevance“ and are then “often regarded as decisive in selection of clinical candidates because of presumptive experimental supremacy. In our view this is specious.” I agree completely! However given the ubiquity of these models regardless of therapeutic modality, it could be helpful to provide additional commentary as to how these models should be appropriately exploited.

Is the topic of the opinion article discussed accurately in the context of the current literature?

Partly

Are arguments sufficiently supported by evidence from the published literature?

Partly

Are all factual statements correct and adequately supported by citations?

Yes

Are the conclusions drawn balanced and justified on the basis of the presented arguments?

Yes

Reviewer Expertise:

Translational research - oncology

I confirm that I have read this submission and believe that I have an appropriate level of expertise to confirm that it is of an acceptable scientific standard.

# Response

**Published:** 2020-12-14

**Authors:** Alexander Kamb

**Affiliations:** A2 Biotherapeutics, USA

1. As part of the “revolution in immune-oncology” seen with checkpoint inhibitors, CAR-T cells, the authors should acknowledge another ‘recombinant cellular therapy’ – oncolytic viruses.
**Our focus is on T-cell therapies, and not intended as an inclusive review. We have added “An opinion” to the title to clarify this. We acknowledge the interest of oncolytic viruses but do not see an unobtrusive way to feather them into our opinion piece.**
2. The authors are appropriately critical of mouse models that “trade off tractability with relevance“ and are then “often regarded as decisive in selection of clinical candidates because of presumptive experimental supremacy. In our view this is specious.” I agree completely! However given the ubiquity of these models regardless of therapeutic modality, it could be helpful to provide additional commentary as to how these models should be appropriately exploited.
**We appreciate this comment, and now include a statement about xenograft models as an example: As a T-cell therapy example, simple xenograft models demonstrate that therapeutic function is compatible with the environment of a mammalian body; nothing more, but nothing less.**

# Peer review: Reviewer response for version 1

**DOI:** 10.5256/f1000research.30072.r74214

**Published:** 2020-11-30

**Authors:** Barbra J. Sasu

**Affiliations:** 1 Allogene Therapeutics, Inc.,, San Francisco, CA, USA

The review deals with an important topic in T cell therapy - the use of appropriate methodology to increase mechanistic understanding and hopefully, eventually, translatability to the clinic.

 At the outset, the authors quote some pioneers in the field of T cell and CAR therapy. Although it’s not possible to add everyone, I would suggest that the addition of Phil Greenberg for his long history of pioneering work on TCR T cells and understanding how to apply engineering to these cells. Perhaps also Mike Jensen and Carl June for understanding the nature of first and second gen CAR T and scientists such as Malcolm Brenner for insights into competitive expansion of CARs in vivo are some suggestions for additions. 

 Analogy between development of SM and T cell therapeutics is an interesting comparison. Text in diagram should perhaps be bigger and QSAR needs to be defined in the legend.

 The paragraph about building PD or mechanistic assays in the same spirit as the SM field is valuable but could be fleshed out more. E.g. The authors comment correctly that most work has to be done with primary cells since Jurkat unable to kill or behave in many other ways like normal T cells. There is reference to heterogeneousness, but perhaps calling out specifically that there is large donor to donor variability would be valuable.

 Adding to in vivo model difficulties I might talk about mouse cytokine environment not supporting human cells without model modification and in syngeneic models, inherent difficulties between human and mouse T cells such as the need for different signaling strengths. 

 When pointing out shortcomings of screening approaches, say it needs to be more mechanistic. Some for instances might be useful, perhaps discussing possibilities for assays that might apply to parts of diagram 2, rather than the traditional endpoints in the T cell field of cytokine secretion, exhaustion markers or killing. Comments that in vivo assay can’t deal with high throughput of candidates is true, but there is the potential for rapid in vivo assay to potentially look at certain aspects covered in diagram e.g. migration, activation, specificity that are hard to cover in vitro. Perhaps a compare on contrast on this would be useful.

 Highlight other potential approaches to increasing tuor specificity e.g. synthetic biology from Wendel Lim or masking would be useful rather than just outlining one approach.

 Quotes that cell therapy doses can exceed 100 billion cells - seems like an outlier and more normal ranges should be quoted both for CARs and TCRs, especially in light of the table comparing CARs and TCRs and in fact cell dose could be added to this table. 

 It was unclear to me if the table is meant to state dogma or the belief of authors. Some of the assumptions already have data challenging them and discussing some of this as a ‘start of the journey’ may be valuable, for example that CARs show good combination with PD-1 Abs in preclinical models.

 At the end, the authors make strong statements that better assays are needed, which I can’t argue with. Perhaps compare and contrast some assays and say what areas merit more development would be good. Potentials for solutions would make this review more valuable and might stimulate some of the general advances in the field that the review calls for.

 The review deals with an important topic in T cell therapy - the use of appropriate methodology to increase mechanistic understanding and hopefully, eventually, translatability to the clinic.

 At the outset, the authors quote some pioneers in the field of T cell and CAR therapy. Although it’s not possible to add everyone, I would suggest that the addition of Phil Greenberg for his long history of pioneering work on TCR T cells and understanding how to apply engineering to these cells. Perhaps also Mike Jensen and Carl June for understanding the nature of first- and second-gen CAR T and scientists such as Malcolm Brenner for insights into competitive expansion of CARs in vivo are some suggestions for additions. 

 Analogy between development of SM and T cell therapeutics is an interesting comparison. Text in diagram should perhaps be bigger and QSAR needs to be defined in the legend.

 Highlight other potential approaches to increasing tuor specificity e.g. synthetic biology from Wendel Lim or masking would be useful rather than just outlining one approach.

 Quotes that cell therapy doses can exceed 100 billion cells - seems like an outlier and more normal ranges should be quoted both for CARs and TCRs, especially in light of the table comparing CARs and TCRs and in fact cell dose could be added to this table. 

 It was unclear to me if the table is meant to state dogma or the belief of authors. Some of the assumptions already have data challenging them and discussing some of this as a ‘start of the journey’ may be valuable, for example that CARs show good combination with PD-1 Abs in preclinical models.

 At the end, the authors make strong statements that better assays are needed, which I can’t argue with. Perhaps compare and contrast some assays and say what areas merit more development would be good. Potentials for solutions would make this review more valuable and might stimulate some of the general advances in the field that the review calls for.

Is the topic of the opinion article discussed accurately in the context of the current literature?

Partly

Are arguments sufficiently supported by evidence from the published literature?

Partly

Are all factual statements correct and adequately supported by citations?

Partly

Are the conclusions drawn balanced and justified on the basis of the presented arguments?

Partly

Reviewer Expertise:

T cell biology, CAR T

I confirm that I have read this submission and believe that I have an appropriate level of expertise to confirm that it is of an acceptable scientific standard, however I have significant reservations, as outlined above.

# Response

**Published:** 2020-12-14

**Authors:** Alexander Kamb

**Affiliations:** A2 Biotherapeutics, USA

At the outset, the authors quote some pioneers in the field of T cell and CAR therapy. Although it’s not possible to add everyone, I would suggest that the addition of Phil Greenberg for his long history of pioneering work on TCR T cells and understanding how to apply engineering to these cells. Perhaps also Mike Jensen and Carl June for understanding the nature of first and second gen CAR T and scientists such as Malcolm Brenner for insights into competitive expansion of CARs in vivo are some suggestions for additions.

**We have mentioned Drs. Greenberg and June in the revision, but ask the reviewer to bear in mind that this is an opinion or perspective, not a review. We have added “An opinion” to the title to clarify this.**

 Analogy between development of SM and T cell therapeutics is an interesting comparison. Text in diagram should perhaps be bigger and QSAR needs to be defined in the legend.

**We have defined QSAR and requested the additional change in size.**

 The paragraph about building PD or mechanistic assays in the same spirit as the SM field is valuable but could be fleshed out more. E.g. The authors comment correctly that most work has to be done with primary cells since Jurkat unable to kill or behave in many other ways like normal T cells. There is reference to heterogeneousness, but perhaps calling out specifically that there is large donor to donor variability would be valuable.

**We have included text to call this variability out specifically: …with considerable donor-to-donor variability;**

 Adding to in vivo model difficulties I might talk about mouse cytokine environment not supporting human cells without model modification and in syngeneic models, inherent difficulties between human and mouse T cells such as the need for different signaling strengths. 

**We have added text to highlight this specific problem (i.e., the mismatch between mouse/human cytokine signaling that can be understood partly as divergence between ligands and receptor pairs over 90 million years of evolutions (e.g., IL-2 ligand and receptor): The human and mouse components of these chimeras, e.g., IL-2 and IL-2R, do not mesh perfectly (Nemoto et al., 1995).**

 When pointing out shortcomings of screening approaches, say it needs to be more mechanistic. Some for instances might be useful, perhaps discussing possibilities for assays that might apply to parts of diagram 2, rather than the traditional endpoints in the T cell field of cytokine secretion, exhaustion markers or killing. Comments that in vivo assay can’t deal with high throughput of candidates is true, but there is the potential for rapid in vivo assay to potentially look at certain aspects covered in diagram e.g. migration, activation, specificity that are hard to cover in vitro. Perhaps a compare on contrast on this would be useful.

**We agree with the reviewer and have pointed out the need to study certain aspects of T-cell biology in vivo, comparing mechanisms that can be studied in vitro with those that require in vivo experimentation. We have added to the legend of Fig. 2 a comment about the need for more mechanistic information. : This diagram illustrates the number and complexity of the steps required to achieve efficacy. Many of these steps can be studied in vitro; for others (e.g., extravasation), in vitro models are inherently problematic.**

 Highlight other potential approaches to increasing tuor specificity e.g. synthetic biology from Wendel Lim or masking would be useful rather than just outlining one approach.

**We have mentioned the SynNotch approach of Dr. Lim and colleagues, (Williams et al., 2020). We have also referenced ligand-binding domain masking approaches and added one reference (Desnoyers et al., 2013): Other approaches are under study, including transcriptional logic circuits and receptor masking (Roybal et al., 2016; Desnoyers et al., 2013). These attempts to widen the target source for selective cancer targets to other targets, including neoantigens and LOH,…**

 Quotes that cell therapy doses can exceed 100 billion cells - seems like an outlier and more normal ranges should be quoted both for CARs and TCRs, especially in light of the table comparing CARs and TCRs and in fact cell dose could be added to this table. 

**We have added a range of T-cell therapeutic doses and altered the sentence: …range from 100 million to 100 billion cells—well beyond the number…**

 It was unclear to me if the table is meant to state dogma or the belief of authors. Some of the assumptions already have data challenging them and discussing some of this as a ‘start of the journey’ may be valuable, for example that CARs show good combination with PD-1 Abs in preclinical models.

**We have changed the title of the legend and added a clause that states: Potential differences among cell therapy targets, receptors, and regulation not yet rigorously tested by mechanistic data. Experiments to test many of these assumptions are underway.**

 At the end, the authors make strong statements that better assays are needed, which I can’t argue with. Perhaps compare and contrast some assays and say what areas merit more development would be good. Potentials for solutions would make this review more valuable and might stimulate some of the general advances in the field that the review calls for.

 The review deals with an important topic in T cell therapy - the use of appropriate methodology to increase mechanistic understanding and hopefully, eventually, translatability to the clinic.

**We make general statements about the kind of assays, but have added text indicating that sensitivity in particular is a useful parameter to measure because it provides a connection among different targets and receptors: In particular, we believe that quantitative assays that measure absolute sensitivity of receptors should be more widely employed, allowing direct comparisons among different targets and receptors.**

# Peer review: Reviewer response for version 1

**DOI:** 10.5256/f1000research.30072.r74217

**Published:** 2020-11-17

**Authors:** John R. James, Muna Fuyal

**Affiliations:** 1 Warwick Medical School, University of Warwick, Coventry, UK; 2 School of Life Sciences, University of Warwick, Coventry, UK

The review article from Kamb and Go provides an overview of how T-cell based therapies are being used in cancer treatment. The challenges arising from this approach and how the foundation of T-cell therapy research can be built for development of more potent treatment of solid tumours are discussed. Their main point is that in the rush to get CAR-T therapy to patients, some of the underlying foundational research has been bypassed, which needs to be ‘filled in’ so the potential benefits of T-cell therapies can be fully realised, which is of course an important concern to raise.

 There are a few points that should be addressed to improve this version of the manuscript:

- The paper would be clearer if the authors could inform about the distinct challenges of CAR-T therapy used for treating blood cancers compared to those of solid tumours. At times, the information is overlapped and slightly unclear.
- In the early discussion of significant players in the CAR-T field, it is remiss not to state the contribution of Carl June’s lab. While much of his group’s work has primarily been in leukaemia rather than solid tumours, it has nonetheless provided real impetus that this approach could be transformational in cancer therapies.
- There is a slight pessimism to the state of knowledge on the mechanism of TCR triggering; while no true consensus will ever be reached on this question, there is little doubt that the fundamental aspects of this signal transduction have been elucidated.
- The authors compare the state-of-the-art development of small-molecule drugs to the equivalent process for T-cell based therapies. They argue that mouse models are not appropriate tools to study (human) immuno-oncology, which is of course strictly true but a charge that can be just as easily levelled at small-molecule drug approaches too and so perhaps unfair for T-cell therapies to be singled out.
- There is no mention of BiTE or ImmTAC therapeutics as alternative T-cell based therapies, which do have potentially greater likelihood of being effective in treating solid tumour masses.
- The authors have listed four additional requirements for effective and safe solid tumour therapy (page 5 under heading Additional challenges for T-cell therapy) along with identifying drug target. The review could do well with more information on these listed points such as current research being carried out to address these limitations.
- There are many labs around the world trying to combine engineering approaches to provide ‘logic-gating’ to CAR-T cell targeting. As the authors state, targets are hard to come by, but the potential for combinatorial CAR-T inputs (AND/NAND/NOT gating) significantly extends the usefulness of some likely targets to more accurately define solid tumour targets. 
- Table 1 describes some commonly-held assumptions about T-cell therapies “not yet rigorously tested by mechanistic data”. The authors do provide a basis for these assumptions but no references to back these up. Whose “commonly held assumptions” are they? 
- The authors state: “This foundation-building work may not be glamorous but is of great consequence and should be valued by scientific journals. If the field as a whole invests to build the infrastructure and expertise of better preclinical models and larger datasets and allocates time to define key mechanistic details prior to clinical testing, we believe the risks required to develop inventive, differentiated therapies will be rewarded with success.” This point should be elaborated on to explain the roles of pharmaceutical companies, scientists and research institutes. Who takes the “unglamourous” job of foundation building? We would argue that academia is taking these risks and doing the foundational work; perhaps the point is aimed more at Pharma that they should also invest more heavily in this work too?

Is the topic of the opinion article discussed accurately in the context of the current literature?

Partly

Are arguments sufficiently supported by evidence from the published literature?

Partly

Are all factual statements correct and adequately supported by citations?

Partly

Are the conclusions drawn balanced and justified on the basis of the presented arguments?

Partly

Reviewer Expertise:

T cell signalling, signal transduction, reductionist approaches, Synthetic biology

We confirm that we have read this submission and believe that we have an appropriate level of expertise to confirm that it is of an acceptable scientific standard, however we have significant reservations, as outlined above.

# Response

**Published:** 2020-12-14

**Authors:** Alexander Kamb

**Affiliations:** A2 Biotherapeutics, USA

**We thank the reviewers for their constructive comments and believe they have understood our key point. We have attempted to address most of the reviewers’ suggestions in the planned revised publication. We point out that we intend our paper to be an opinion or perspective, and not a review. Consequently, we have limited some of the references and discussion.**

- The paper would be clearer if the authors could inform about the distinct challenges of CAR-T therapy used for treating blood cancers compared to those of solid tumours. At times, the information is overlapped and slightly unclear.
- **We agree and have added text to clarify the specific challenges of solid tumors. Perhaps most dramatically, infused T-cell therapeutics directed against solid tumors must extravasate to reach their targets, targets that may be present on a subset of vital normal tissues as well.  **
- In the early discussion of significant players in the CAR-T field, it is remiss not to state the contribution of Carl June’s lab. While much of his group’s work has primarily been in leukaemia rather than solid tumours, it has nonetheless provided real impetus that this approach could be transformational in cancer therapies.
- **We agree and have included Dr. June’s name and referenced his contributions. Still others have made substantive contributions to understanding, design and development of next-generation CAR-Ts; for example, C. June and P. Greenberg (see for review Guedan et al., 2019).**
- There is a slight pessimism to the state of knowledge on the mechanism of TCR triggering; while no true consensus will ever be reached on this question, there is little doubt that the fundamental aspects of this signal transduction have been elucidated.
- **We do not intend pessimism, and have clarified our view that, though important basic mechanistic questions remain (e.g., altered-peptide ligands, APLs), the TCR and CAR signaling mechanisms are understood in outline at least (a good example is the work of Dr. James): For example, there is not a broadly accepted model that explains key behavior of TCRs with respect to sensitivity and selectivity toward their ligands, peptide major histocompatibility complexes (pMHCs). CAR signaling, though understood in outline, also lacks important details (see for review Courtney *et al.*, 2018; Nerreter *et al.*, 2019).**
- The authors compare the state-of-the-art development of small-molecule drugs to the equivalent process for T-cell based therapies. They argue that mouse models are not appropriate tools to study (human) immuno-oncology, which is of course strictly true but a charge that can be just as easily levelled at small-molecule drug approaches too and so perhaps unfair for T-cell therapies to be singled out.
- **We agree wholeheartedly and have clarified this point: These deficits apply to small- and large-molecule therapeutic discovery.**
- There is no mention of BiTE or ImmTAC therapeutics as alternative T-cell based therapies, which do have potentially greater likelihood of being effective in treating solid tumour masses.
- **We know these modalities well, but believe that cell therapy holds more promise for solid tumor therapies. Cells can be engineered, if they do not do so already, to distribute into tissues. Large molecules (soluble proteins) are much more limited in what they can be engineered to do, beyond binding things, and are constrained by their physico-chemical properties. **
- The authors have listed four additional requirements for effective and safe solid tumour therapy (page 5 under heading Additional challenges for T-cell therapy) along with identifying drug target. The review could do well with more information on these listed points such as current research being carried out to address these limitations.
- **We should be clear that we do not intend to review the topic; our publication is more properly classified as an opinion piece. These topics are beyond the scope of our paper, and there are numerous reviews in the literature.**
- There are many labs around the world trying to combine engineering approaches to provide ‘logic-gating’ to CAR-T cell targeting. As the authors state, targets are hard to come by, but the potential for combinatorial CAR-T inputs (AND/NAND/NOT gating) significantly extends the usefulness of some likely targets to more accurately define solid tumour targets. 
- **We have added a sentence to emphasize this point; i.e., that there are other logic systems beyond the AND NOT logic we describe in brief: Other approaches are under study, including transcriptional logic circuits and receptor masking (Roybal et al., 1995; Desnoyers et al., 2013). These attempts to widen the target source for selective cancer targets to other targets, including neoantigens and LOH…**
- Table 1 describes some commonly-held assumptions about T-cell therapies “not yet rigorously tested by mechanistic data”. The authors do provide a basis for these assumptions but no references to back these up. Whose “commonly held assumptions” are they? 
- **We encounter people with different subsets of these assumptions frequently, but it is difficult to provide a suitable reference. We have changed the wording of the Table 1 title and in the text: There are many potential differences between, for example, TCRs and CARs which have not been tested systematically, and the field would benefit from their thorough examination (Table 1). We are certainly open to alternative phrasing. **
- The authors state: “This foundation-building work may not be glamorous but is of great consequence and should be valued by scientific journals. If the field as a whole invests to build the infrastructure and expertise of better preclinical models and larger datasets and allocates time to define key mechanistic details prior to clinical testing, we believe the risks required to develop inventive, differentiated therapies will be rewarded with success.” This point should be elaborated on to explain the roles of pharmaceutical companies, scientists and research institutes. Who takes the “unglamourous” job of foundation building? We would argue that academia is taking these risks and doing the foundational work; perhaps the point is aimed more at Pharma that they should also invest more heavily in this work too?

**We strongly agree that there should be investment in foundation-building academic research by granting agencies, and have added this opinion explicitly: Our strong view is that granting agencies should invest in foundation-building academic research, in part because shorter-term translational work is often attractive to the private sector.**
